# Fatigue Behavior of Heavy-Haul Railway Prestressed Concrete Beams Based on Vehicle-Bridge Coupling Vibration

**DOI:** 10.3390/ma15082923

**Published:** 2022-04-16

**Authors:** Li Song, Ran Liu, Chenxing Cui, Zhiwu Yu, Wenchang Zhang

**Affiliations:** 1 School of Civil Engineering, Central South University, Changsha 410075, China; songli@csu.edu.cn (L.S.); cui-chx@csu.edu.cn (C.C.); zhwyu@csu.edu.cn (Z.Y.); zhangwenchang8@163.com (W.Z.); 2 National Engineering Research Center of High-Speed Railway Construction Technology, Changsha 410075, China

**Keywords:** fatigue flexural behavior, prestressed concrete beams, heavy-haul railway, fatigue behavior assessment model, fatigue life

## Abstract

Due to the demand for increasing trainload and enhancing some existing heavy-haul railways, the low reserve value of bearing capacity is a problem for the 32 m-span simply supported beam. The fatigue behavior of prestressed concrete beams in a heavy-haul railway loaded by 33 t and larger axle weight of trains was experimentally investigated. The experimental results of the fatigue behaviors, including fatigue deformation, crack propagation behavior, and strains of classical materials were obtained and analyzed. A fatigue behavior assessment model was established to investigate the residual stiffness and yield point degradation of the beams loaded by the trainload. The effects of train fatigue cycles and prestress loss on the residual stiffness and yield point degradation models of the beams were analyzed. The results indicated that the crack development process had three stages during the fatigue process: the derivative stage, gradual development stage, and fatigue failure stage. Trainload was the main external factor influencing the fatigue behavior of prestressed concrete beams. The increase in trainload accelerated the degradation rate of the residual stiffness of the beams and yield point, reducing the fatigue life. The prestressing strand was primarily used to delay the concrete cracking in the tension zone. When the beam was not cracked, the prestressed concrete beam showed good fatigue performance, and the degree of prestressing did not affect the fatigue life of the beams. When the maximum fatigue load exceeded the cracking load of the beam, prestress loss in beams became a critical issue that accelerated the degradation rate of fatigue strength and reduced fatigue life. The higher the fatigue damage degree, the more pronounced the effect of prestress loss on the fatigue strength of the beams. The fatigue failure of prestressed concrete beams occurred in the bottom tensile steel bar. Therefore, when the trainload of a heavy-haul railway is greater than the cracking load of the beam, it is recommended to strengthen the beam by prestressing and strictly control the trainload to avoid yield failure.

## 1. Introduction

Prestressed concrete structures are widely used in essential facilities such as buildings, bridges, and offshore platforms due to the advantages of a lightweight, high stiffness, and good crack resistance. In China, fully prestressed concrete beams are widely used in heavy-haul railway bridges [1]. For example, a heavy-haul railway has 1583 standard simply supported beams on the whole line, of which prestressed concrete beams account for more than 95% [2]. To solve the problem of transportation capacity, China plans to enhance some existing heavy-haul railways and increase the axle weight of heavy-haul trains from 21 t–25 t to 30 t or more. Static analysis shows that the reserve values of bearing capacity for 24 m and 32 m span simply supported beams are 1.16 (1.10) and 1.12 (0.96), respectively, under the 30 t (33 t) axle weight of trains [3]. Thus, under the combined effect of multiple factors such as train loads, concrete damage deterioration, and prestress loss, fully prestressed concrete beams are faced with a significant risk of fatigue cracking [4,5,6,7,8]. Fatigue cracking will affect the service life of beams, and even affect driving safety in the long term [9,10]. Therefore, it is urgent to investigate the fatigue performance of prestressed concrete beams under the axle weight of heavy-haul trains, supporting the maintenance of heavy-haul railway bridges.

Several studies have been performed on the fatigue performance of prestressed concrete beams. Castilho et al. [11,12,13,14] conducted a constant amplitude fatigue load on partially prestressed concrete beams, and the results showed that fatigue failure generally starts from the fatigue fracture of the outermost tensile steel bars. Ren et al. [15] studied the stiffness degradation law of prestressed concrete beams after fatigue loading through experiments and found that the residual displacement of beams increased gradually with the fatigue cycles and showed a three-stage law. Huszár et al. [16] studied the section crack resistance of prestressed concrete beams, and the influence of flexural tensile fatigue strength of concrete was analyzed. Then, the S-N curve of the section crack resistance of prestressed concrete beams was proposed under constant-amplitude cyclic loading. Fatigue can cause concrete cracking and corrosion of tendons along bridge girders, resulting in significant prestress loss and deflection during the service life. Kashani et al. [17,18] studied the adverse effect of corrosion on ductility, flexural, shear, and axial capacity loss of the corroded reinforced concrete components through experiments, and found that corrosion has a much more adverse impact on the ductility of the reinforced concrete columns than strength. However, the effect of corrosion on ductility and strength reduction of reinforced concrete beams was the same. Bonopera et al. [19] proposed a novel method for identifying residual prestress force in simply supported prestressed concrete girder bridges. When information on the flexural stiffness of the beam is known, the method can estimate the prestress force by measuring the vertical deflection at the quarter or the midspan of the prestressed concrete girder bridge.

Compared with general highway and railway bridges, the applied load of heavy-haul railway bridges has the characteristics of large load, large-amplitude vibration, high frequency, etc., which makes the cumulative fatigue damage and cracking problems more prominent. Yu et al. [20,21] studied the fatigue behavior and crack propagation laws of heavy-haul railway bridges through experiments, and found that the beams will have excellent fatigue resistance when the bottom concrete is compressed under the maximum fatigue load. However, when the fatigue load level is higher than the cracking load, the beams will show poor crack resistance, decreasing the fatigue life. Once the cracking occurs, the fatigue damage characteristics of partially prestressed concrete beams will be observed, and concrete cracks will accelerate the fatigue fracture of steel bars. If the load level causes the beams to crack, heavy-haul railway prestressed concrete beams will be at great risk of fatigue failure. Therefore, it is urgent to deeply examine heavy-haul railway prestressed concrete beams’ fatigue damage and failure characteristics. Analysis methods of prestressed concrete beams mainly include the empirical model (S-N curve), fatigue crack growth model based on fracture mechanics, and fatigue performance analysis model based on damage mechanics. Existing research mainly focuses on the former two analysis methods [22,23]. Firstly, in the S-N curve method, the stress and strain of constituent materials can be obtained through static and dynamic analysis of the beams, based on which the fatigue life can be estimated. However, the results obtained by this method are not universal, and this method cannot reveal the evolution process of structural fatigue damage. Secondly, the fatigue crack growth method uncovers the physical mechanism of the fatigue problem to a certain extent, which dramatically advances the understanding of the fatigue problem [24]. Nevertheless, this method is more suitable for describing single macro fatigue crack propagation. Considering the complexity of concrete structures, the development of continuum damage mechanics provides a new approach to fatigue analysis of prestressed concrete beams.

The existing literature only presented limited information on the fatigue performance of heavy-haul railway prestressed concrete beams. According to the demand for increasing trainload and enhancing some existing heavy-haul railways, the low reserve value of bearing capacity is a problem for the 32 m-span simply supported beam. In this study, standard 32 m prestressed concrete beams in the heavy-haul railway were tested under fatigue loading, and the fatigue performance of prestressed concrete beams under 33 t or higher trainload was examined. A numerical model based on the fatigue properties of materials and Vehicle-Bridge coupling vibration was proposed to assess the fatigue behavior of the field beams. The fatigue performance assessment model of the field beams was verified with the experimental results. The presented model was then employed to study the effect of fatigue load range and prestress loss on fatigue life, fatigue strength, stiffness, and post-fatigue ultimate capacity. 

## 2. Experimental Program

### 2.1. Materials and Specimens

**Figure 1:**
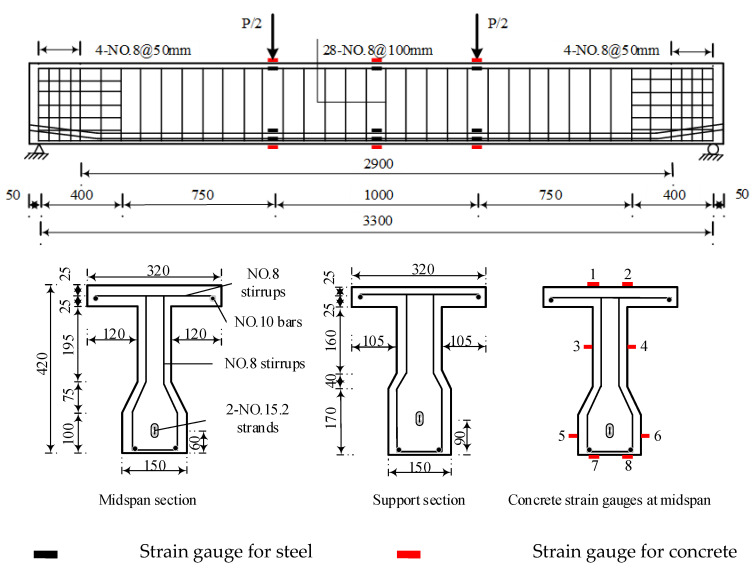
Details of the test specimens (unit: mm).

**Figure 2:**
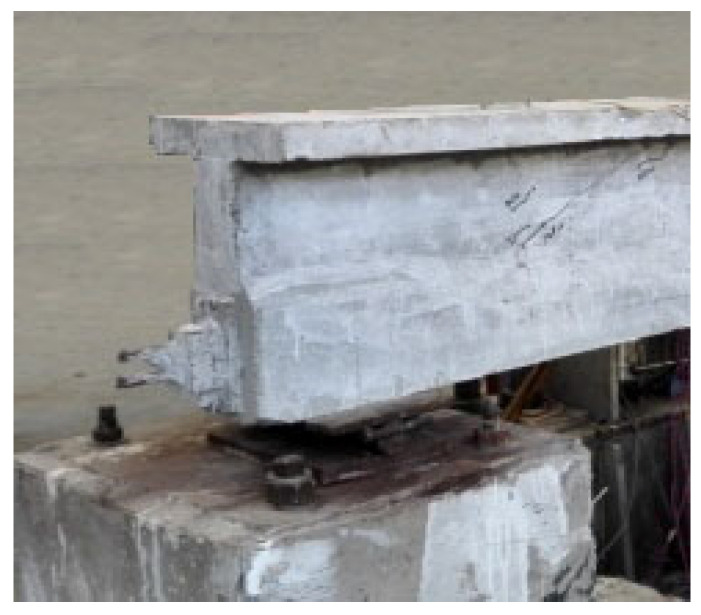
Anchor end of the model beam.

Standard 32 m prestressed concrete beams in heavy-haul railway were taken as the prototype, and five scale test model beams were designed. The geometry and reinforcement of the model beams are shown in Figure 1. Six concrete cubes and six prisms were taken as the test blocks for each test beam. The mixture proportions and type of concrete are shown in Table 1. The concrete strength was tested at three time periods, and the average compressive strength value was 53.6 MPa. Three 400 mm long samples were taken for each steel bar type to determine the mechanical properties of tensile steel bars and stirrups, as shown in Table 2. Two bundles of 7Φ^s^5 steel strands (nominal diameter: 15.2 mm) were selected as prestressing steel bars with an ultimate tensile strength value of *f*_pk_ = 1860 MPa and parabolic shape. The steel strands were tensioned at one end, and the concrete age had exceeded 28 days. The beam ends were equipped with reinforcement mesh, an embedded steel plate, and spiral reinforcement to withstand the local pressure. The anchor end of the model beam as shown in Figure 2. The prestress was divided into five levels and tensioned to 1436 MPa (controlled tensile stress is *σ*_con_ = 1395 MPa). The initial prestress was about 77% of the ultimate tensile strength. 

### 2.2. Test Procedures

**Figure 3:**
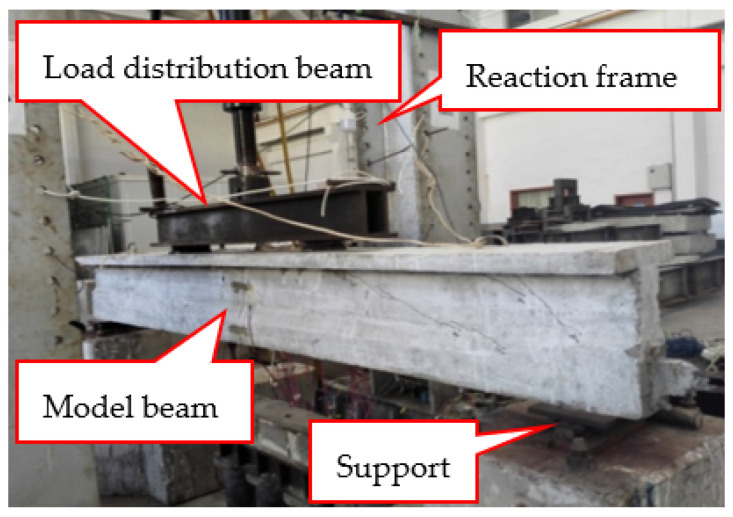
Test setup for the model beam.

The test setup for the model beam is shown in Figure 3. The layout of the strain gauges on the tensile steel bars and prestressed steel strands is shown in Figure 1. During static loading and unloading after fatigue cycles, three displacement sensors were used to measure the displacement of the test beams at the midspan and the loading points. Two displacement sensors were used to measure the support settlement of the test beams. When cracks were found in the beams, statistics were obtained on their width, length, and position.

The loading conditions of the test beams are shown in Table 3. Specimen S1 was used for the static loading test. Specimen C1 was loaded to failure by static load after 3.05 million times fatigue cycles, and specimens C2, C3, and C4 were used for the constant-amplitude fatigue loading test. The minimum fatigue load *P*_min_ was determined according to the dead load of heavy-haul railway bridges. Then, under the action of gravity and *P*_min_, the tensile steel stress of the model beam was the same as that of the field beam under the dead load. The maximum fatigue load *P*_max_ of the C1 beam was determined according to the stress amplitude of the tensile reinforcement of 32 m prestressed concrete beam analyzed in Section 3.1 under 33 t trainload. Then, the stress amplitude of the tensile steel bar of the C1 beam under the action of *P*_min_ and *P*_max_ was consistent with that of the original beam. The C2–C4 beams were used to investigate the fatigue behavior of prestressed concrete beams when the trainload continues to increase to exceed the cracking load. 

The tests were performed on a PMS-500 electo-hydraulic servocontrolled fatigue testing machine. The loading frequency of the testing machine is 0–5 Hz. For the fatigue test, the test beams were loaded to the maximum fatigue load first, and then the applied load was reduced to the mean value. After that, a sine wave load cycle was applied to the test beams, and the loading frequency was 3 Hz. The fatigue tests were completed at the predetermined number of cycles, and a static loading test was performed to obtain the displacements and the strains.

### 2.3. Test Results

#### 2.3.1. Static Performance

**Figure 4:**
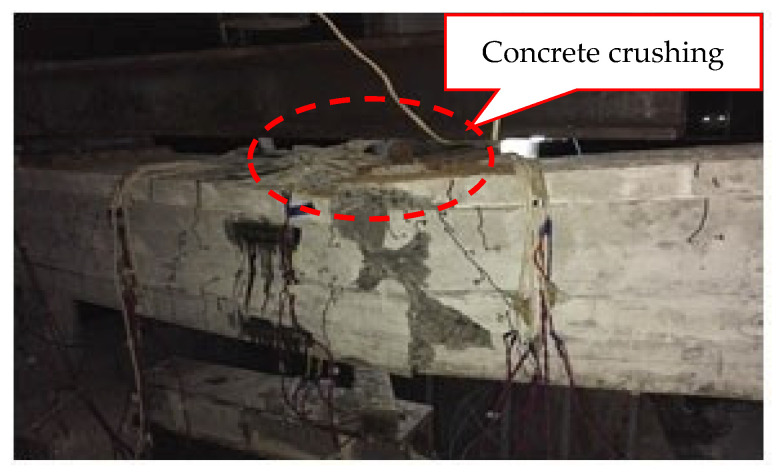
The failure mode of the test beam S1.

**Figure 5:**
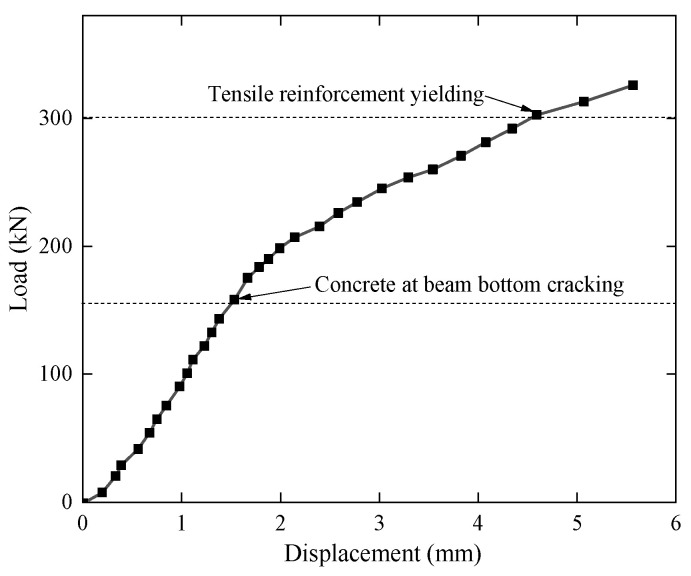
The load-displacement curve of the beam under static loading.

**Figure 6:**
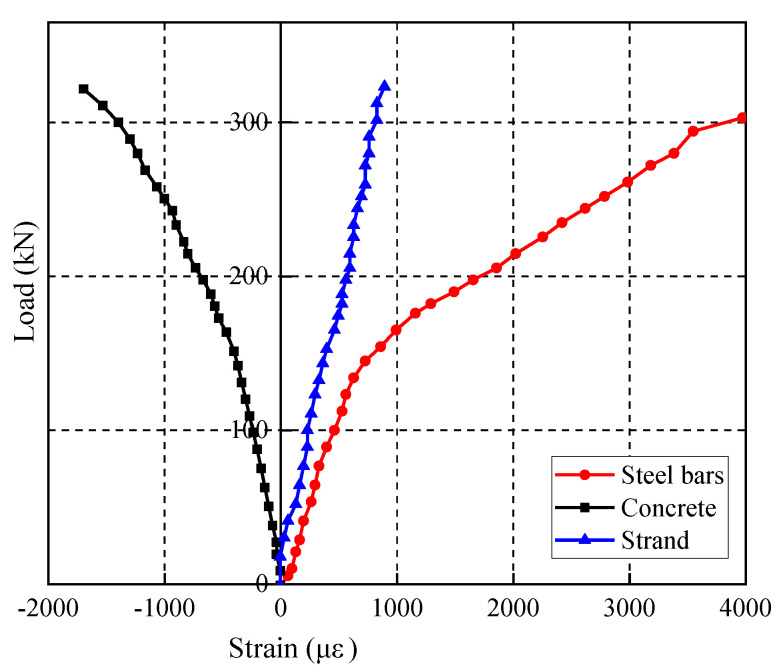
Load-strain curves of the beam under static loading.

The tensile steel bar of the test beam S1 under static loading yielded first. Then, the concrete in the compression zone began to crush. The collapse of the mode beam was governed by concrete crushing, and the failure mode is shown in Figure 4. When the concrete at beam bottom cracked, and the concrete at beam top crushed after tensile steel bar yield, the applied loads were 157.0 kN and 324.2 kN, respectively. Figure 5 shows the load-midspan displacement curve of the test beam S1 under static loading. The load-strain curves of concrete in the compression zone, prestressing strand, and tensile steel bar are shown in Figure 6, where a negative strain indicates compression, and a positive strain indicates tension. 

It should be noted that after the prestressing strands were tensioned, the strain of the prestressing strand in the midspan of the beam is about 4762 με, the strain of the tensile steel bars at the bottom of the beam is about −342 με. Then, the high-pressure grouting pump is used to grout and seal from the reserved grouting port. Due to the reasons for construction, some strain gauges of steel bar and prestressing strand were damaged before or at the beginning of the test. Therefore, in the result analysis, the undamaged strain data in the flexural segment were selected as far as possible. The strain of the concrete compression zone was given by strain gauge 1 illustrated in Figure 1. In the midspan of the beam, the strain of the prestressed steel strand was given by the outermost prestressed steel strand and the strain of the steel bar was given by the outermost tensile steel bar at the bottom. The influence of effective prestress and weight of the test beam was not considered before static loading, and the initial strain and the initial displacement were zeroed. It can be seen from the figure and the test phenomenon that after the concrete at beam bottom cracked, the increase rate of strain and midspan displacement of the test beam all increased significantly. When the applied load was 300 kN, the strain of the tensile steel bar changed abruptly; when the applied load reached 324 kN, the concrete in the compression zone was crushed.

#### 2.3.2. Fatigue Failure Modes 

**Figure 7:**
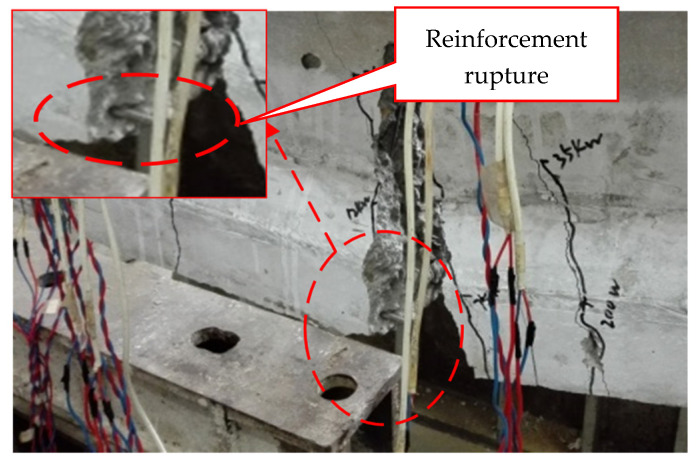
The fatigue failure mode of the test beam.

The model beams test showed that when the fatigue load level was lower than the pre-compression stress of concrete in the tension zone, the test beams exhibited excellent fatigue performance. With the increase in fatigue load level, the fatigue damage characteristics of the test beams were similar to those of partially prestressed concrete beams with a high prestressing degree [21]. The fatigue failure of those of test beams occurred in the bottom tensile steel bar. Further loading caused the height and width of the main fatigue crack to increase rapidly, and the fatigue fracture of the remaining ordinary steel bar occurred successively. At this stage, the fatigue failure of prestressing strand and concrete in the compression zone did not occur (their average maximum strains are less than 1000 µε and 9000 µε, respectively). However, there were many vertical cracks in the pure flexural segment of the beams, and the deformation of the beams increased sharply. Consequently, the maximum crack width and deformation could not meet the requirements of the specification. The fatigue failure mode of the beam is shown in Figure 7. Therefore, it is more reasonable to take the number of fatigue fracture cycles of the bottom tensile steel bars as the fatigue life of the beams.

#### 2.3.3. Development of the Displacement and Main Crack

**Figure 8:**
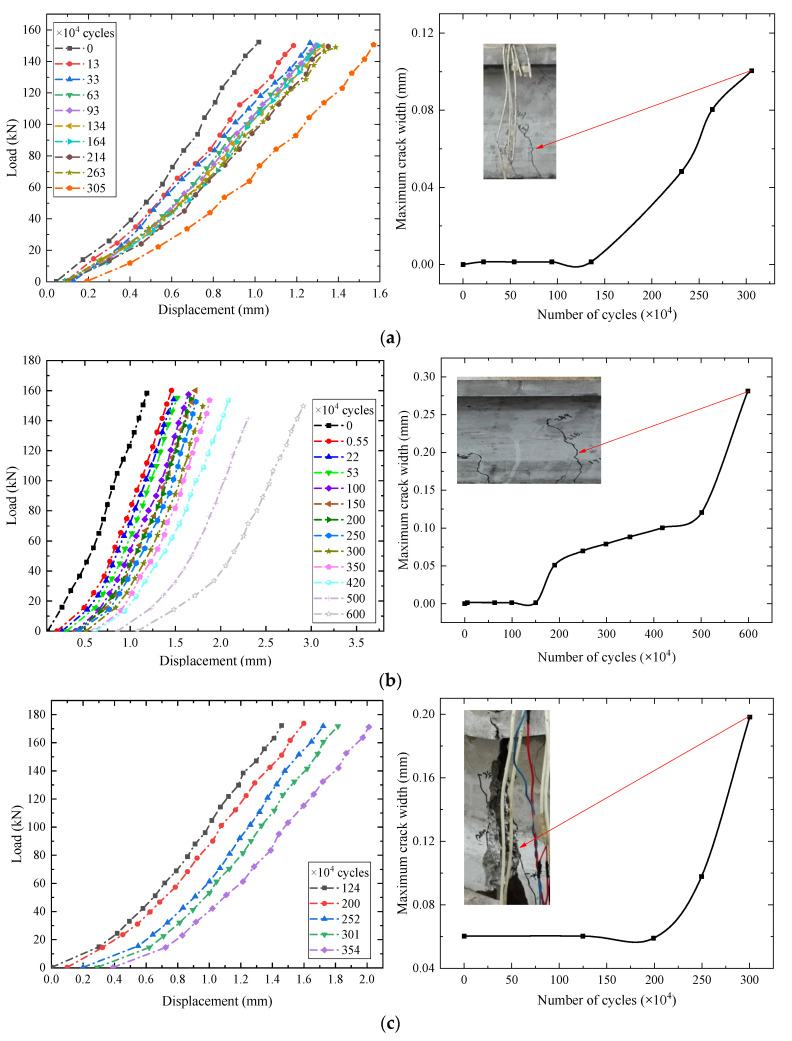

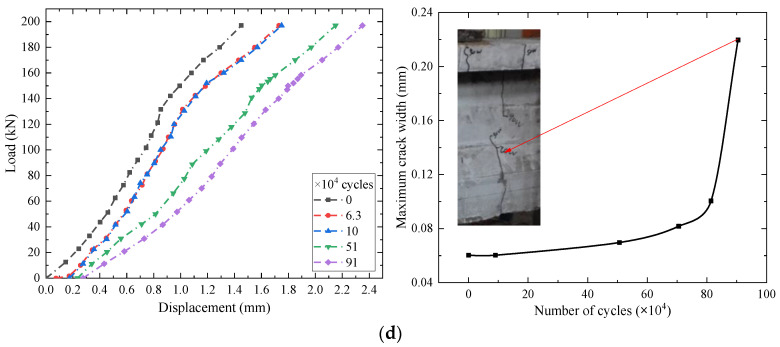
Development of the midspan displacement and main crack under fatigue loading: (**a**) The test beam C1; (**b**) The test beam C2; (**c**) The test beam C3; (**d**) The test beam C4.

**Figure 9:**
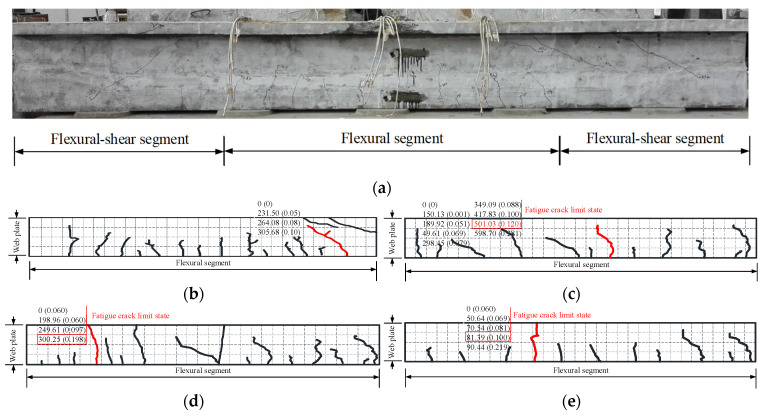
Crack distributions of the test beams (unit: mm): (**a**) General view (C1); (**b**) The test beam C1; (**c**) The test beam C2; (**d**) The test beam C3; (**e**) The test beam C4.

Figure 8 shows the relationship curves between the maximum crack width and the number of fatigue cycles of C1–C4 test beams. It can be seen from the figure that during the fatigue process, the crack development process had three stages: derivative stage, gradual development stage, and fatigue failure stage. The crack width in the derivative stage was about 0 mm–0.06 mm. In the gradual development stage, during the fatigue loading process, the number, length, and width of cracks in the pure flexural segment of the beams were constantly developing (see Figure 9). The number shown in Figure 9 represents the loading cycles (unit: ×10^4^) and main crack width (unit: mm). For instance, 249.61 (0.09) indicates that the main crack (red crack) of the test beam C3 width is 0.09 mm after 249.61 × 10^4^ fatigue cycles, and the main crack width in the gradual development stage was about 0.06–0.10 mm. When the maximum crack width of the test beams C2, C3, and C4 reached 0.10 mm, the corresponding fatigue cycles were 4.17 million cycles, 2.6 million cycles, and 0.81 million cycles, respectively. Throughout the whole fatigue test, the damage of the test beams accumulated, and the cracks expanded due to cyclic creep. When the stress of the bottom tensile steel bar reached the yield point, the displacement of the beams and the width of the main crack increased rapidly with the fatigue cycles, which eventually led to the fatigue failure. Compared with partially prestressed concrete beams [21], fully prestressed concrete beams are equipped with only a small amount of tensile steel bars, which is more sensitive to cracks. Once cracked, the bottom tensile steel bars of the beam can easily undergo fatigue failure. Hence, the cracking of fully prestressed concrete beams should be strictly controlled.

**Figure 10:**
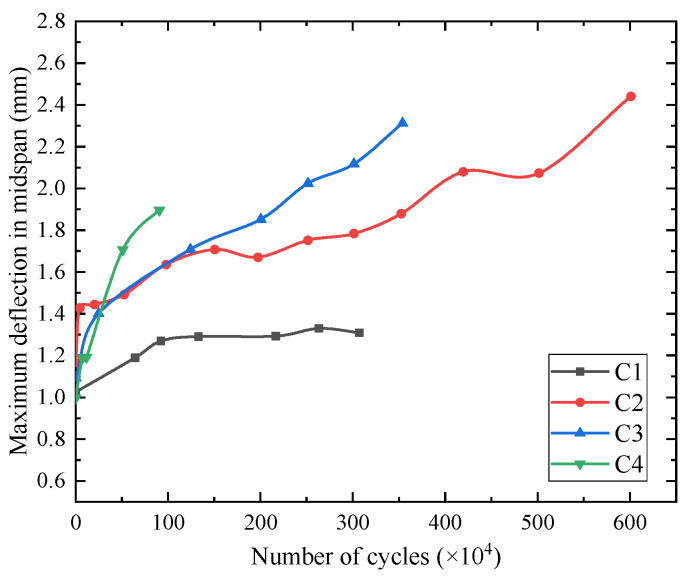
The relationship between the number of cycles and midspan displacement.

**Figure 11:**
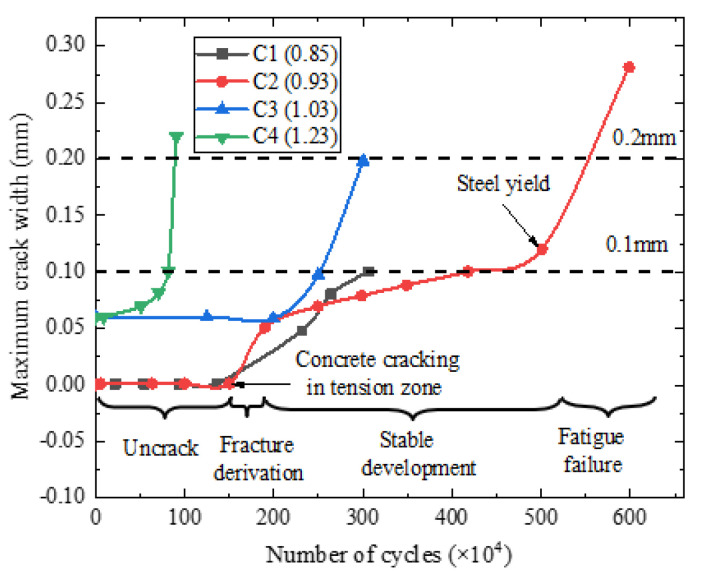
The relationship between the number of cycles and the fatigue main crack width.

The relationship between the number of fatigue cycles and midspan displacement of the beams is shown in Figure 10, and the relationship between the number of fatigue cycles and the fatigue main crack width is shown in Figure 11. The average increase speed of midspan displacement is defined as *y*/*n* (unit: ×10^−6^), where *y*/*n* is the ratio of midspan displacement *y* (unit: mm) and the number of cycles *n*. The fatigue crack limit state is a critical indicator of prestressed concrete structures. It can be seen from Figure 10 and Figure 11 that when the cracking coefficients of the test beams were 0.93, 1.03, and 1.23, the fatigue loading cycles were 4.17 million, 2.6 million, and 0.81 million, respectively. Moreover, the maximum crack width reached 0.10 mm, exceeding the limit value specified in the Chinese standard GB 50010-2015 [25]. It indicates that prestressed concrete beams reached the fatigue crack limit state. The corresponding maximum displacements were 2.079 mm, 2.043 mm, and 1.862 mm. The maximum displacement and span ratio was 1/1772, which is less than the limit (1/800) specified in the code [26]. Before entering the fatigue failure stage, the average increase rates of the midspan displacement of the test beams C2, C3, and C4 were 0.498, 0.786, and 2.298, respectively. By comparison, it can be inferred that the higher the load level, the faster the stiffness degradation of the beams.

#### 2.3.4. Development of Strain

**Figure 12:**
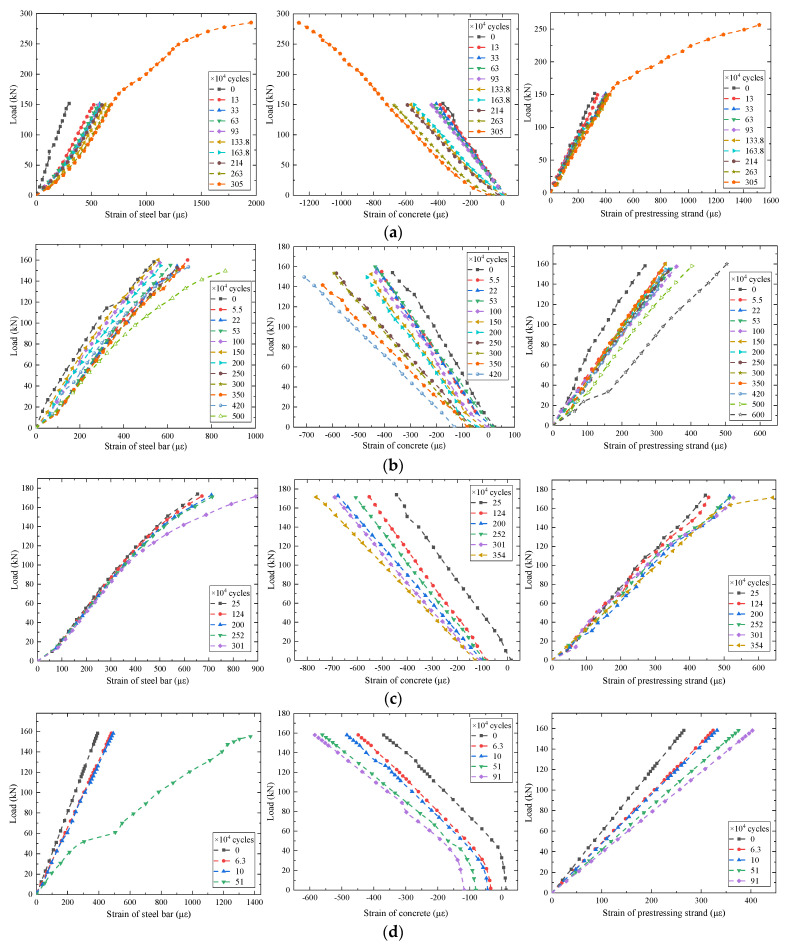
Strain development of concrete, steel bar, and prestressing strand under constant-amplitude fatigue loading: (**a**) The test beam C1; (**b**) The test beam C2; (**c**) The test beam C3; (**d**) The test beam C4.

The load-strain curves of concrete in the compression zone, prestressing strand, and tensile steel bar of the beam are shown in Figure 12, where a negative strain indicates compression and a positive strain indicates tension. The strain of the concrete compression zone was given by strain gauge 1 illustrated in Figure 1. In the midspan of the beam, the strain of the prestressed steel strand was given by the outermost prestressed steel strand and the strain of the steel bar was given by the outermost tensile steel bar at the bottom. The effect of effective prestress and weight of test beam on the initial strain of concrete before testing was considered, the initial value of each load–strain curve of steel bar and the prestressing strand was zeroed. The test results show that with an increasing number of fatigue cycles, the residual strain of compression zone concrete increases gradually and the slope of the load-strain curves decreases continuously. In addition, it can also be observed that the slope of load–strain curves of tensile steel bar and the prestressing strand was continuously decreasing. Due to the damage of strain gauge, parts of strain data in the final stage of fatigue were not obtained.

## 3. Modelling 

### 3.1. Models

#### 3.1.1. Fatigue Life Models of Steel and Concrete

##### Fatigue Life of Steel Bar

The relationship between the stress amplitude of the tensile steel bar and the number of fatigue failure cycles (fatigue life) is defined by the S-N curve (see Equation (1)).

(1) $$N=C{\left(\Delta \sigma \right)}^{-m}$$

where ∆*σ* is the fatigue stress amplitude, *N* is the number of constant-amplitude fatigue failure cycles, *m* is the material constant, and *C* is the fatigue detail constant of the tensile steel bar.

**Figure 13:**
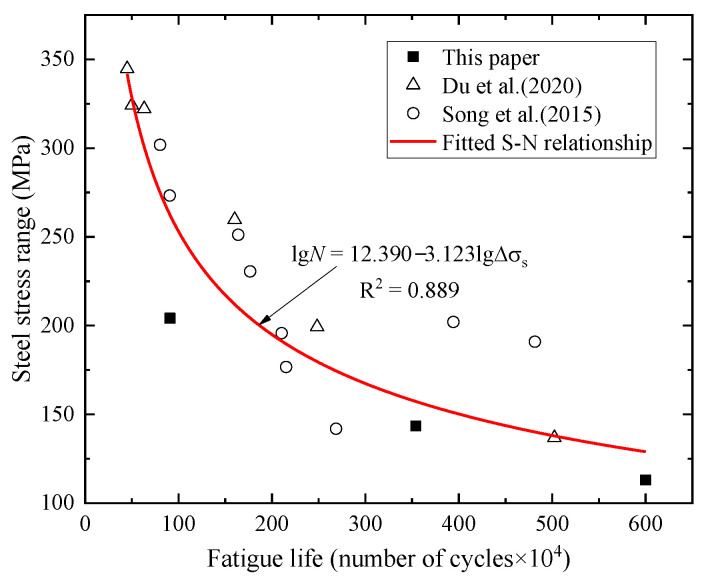
The relationship between the stress amplitude of the steel bar and the number of fatigue failure cycles [21,27].

It can be seen from the above analysis that the fatigue failure of prestressed concrete beams occurs in the outermost tensile steel bars. Table 3 lists the fatigue life of the test beams under various fatigue load levels, and Figure 13 shows the fatigue stress amplitude and the fatigue life data points of the bottom tensile steel bars of the test beams within the fatigue loading range. An S-N curve was regressed using the experimental data points and the existing research data of similar cases [21,27]. Chinese standard TB 10092-2017 [28] defines that the stress range corresponding to 2 million cycles under constant amplitude cyclic loading is the fatigue strength in the S-N curve. The allowable fatigue stress amplitude for steel bars is 195.106 MPa, ensuring that the fatigue life is greater than 2 million cycles.

(2) $$\mathrm{lg}N=12.390-3.123\mathrm{lg}\Delta \sigma_{s}$$

For the fatigue life of prestressing strand, Naaman [13] conducted a large number of relevant tests, and a representative S-N curve equation was given as follows:

(3) $$f_{r}/f_{\mathrm{pt}}=-0.123\mathrm{lg}N+0.870$$

where *f*_r_ and *f*_pt_ are the stress amplitude and the ultimate tensile strength, respectively.

The fatigue damage process of the steel bar is a crack propagation process and reduces the effective bearing area [29]. This paper uses the reduction in the effective bearing area as the characterization factor for the fatigue damage of the steel bar. The initial effective bearing area of the tensile steel bar and prestressing strand before the fatigue loading of prestressed concrete beams are represented as *A*_s(p)_. When the tensile steel bars or prestressing strand reach the fatigue limit state after fatigue loading *N* cycles, the effective bearing area is *A*_s(p)_(*N*). The force in the steel bars before and after fatigue loading should satisfy the following formula:

(4) $$A_{s\left(p\right)}^{}\sigma_{s\left(p\right),\;\max}^{}=A_{s\left(p\right)}^{}(N_{s\left(p\right)}^{})f_{\mathrm{ys}(p)}$$

where the subscript s and p represent the tensile steel bar and prestressing strand, respectively, *f*_ys(p)_ is the yield strength of the tensile steel bar and prestressing strand.

It is assumed that the effective bearing area of the steel bars under constant-amplitude fatigue stress conforms to the Miner linear cumulative damage criterion [18]. The effective bearing area of the tensile steel bar or prestressing strand after fatigue loading *n* cycles can be expressed as:

(5) $$A_{s\left(p\right)}^{}(n)=A_{s\left(p\right)}^{}(1-\frac{n}{N}(1-\frac{\sigma_{s\left(p\right),\;\max}^{}}{f_{\mathrm{ys}(p)}}))$$

The tensile stress of the tensile steel bar is greater than the yield stress, and the tensile stress of prestressing strand is greater than the ultimate tensile strength.

##### Concrete Constitutive Model and Fatigue Life

A previous study [30] proposed a fiber bundle irrecoverable (strain) chain model based on the classical parallel element model to express the damage characteristics of concrete materials. It then established the static damage constitutive model of concrete was established as follows:

(6) $$\left\{\begin{array}{l} \sigma =(1-D):E_{0}:\left(\varepsilon -\varepsilon^{P}\right)\\ \varepsilon =\varepsilon^{e}+\varepsilon^{P} \end{array}\right.$$

where *σ* is the stress tensor; *E*_0_ is the initial elastic stiffness tensor of concrete; *D* is the concrete damage tensor; *ε* is the concrete strain tensor; *ε*^e^ and *ε*^p^ are the concrete elastic strain tensor and plastic strain tensor, respectively; “:” is the double dot product.

(7) $$\bar{\sigma }=E_{0}:\left(\varepsilon -\varepsilon^{P}\right)$$

(8) $$\sigma =(1-d^{\pm }){\bar{\sigma }}^{\pm }$$

where ${\bar{\sigma }}^{\pm }$ is the tensile (compressive) effective stress of concrete, and $d^{\pm }$ is the tensile (compressive) damage variable of concrete, which is calculated according to Equation (9) [31]:

(9) $$\left\{\begin{array}{l} d^{+}=A_{1}^{+}+(A_{2}^{+}-A_{1}^{+})\left[\frac{c}{1+10^{p_{1}\cdot {(x}_{1}-x)}}+\frac{1-c}{1+10^{p_{2}\cdot {(x}_{2}-x)}}\right]\\ d^{-}=\frac{A_{1}^{-}-A_{2}^{-}}{1+{\left(x/x_{0}\right)}^{p_{3}}}+A_{2}^{-} \end{array}\right.$$

where $x=\frac{\varepsilon }{\varepsilon_{k}^{\pm }}$, $\varepsilon_{k}^{\pm }$ is the peak tensile (compressive) strain of concrete corresponding to the tensile (compressive) strength. *A*_1_^+^, *A*_2_^+^, *P*_1_, *P*_2_, *c*, *x*_1,_ and *x*_2_ are parameters related to *d*^+^, *A*_1_^−^, *A*_2_^−^, *P*_3_, and *x*_0_ are parameters related to *d*^−^. These parameter values can be obtained from the literature [32].

Based on the damage characteristics of concrete under static loading and the influence of repeated loading on the evolution of concrete damage, the static beam-chain model was extended to the fatigue beam-chain model in the literature [32] to express the mechanical behavior of concrete under repeated loading. Then, the constitutive model of concrete after fatigue damage was proposed:

(10) $$\sigma_{n}=(1-d_{n}^{\pm }){\bar{\sigma }}_{n}^{\pm }$$

where ${\bar{\sigma }}_{{}^{n}}^{\pm }$ is the tensile (compressive) effective stress of concrete after *n* fatigue recycles, and $d_{n}^{\pm }$ is the tensile (compressive) damage variable of concrete after *n* fatigue recycles, which is calculated according to Equation (11):

(11) $$d_{n}^{\pm }=d_{1}^{\pm }+d_{0}^{\pm }\cdot (d_{N}^{\pm }-d_{1}^{\pm })$$

where $d_{0}^{\pm }$ is the concrete fatigue damage variable after *n* fatigue recycles, calculated according to Equation (12). $d_{1}^{\pm }$ and $d_{N}^{\pm }$ are the concrete fatigue damage variables after the first and *N*th repeated loading, respectively, and calculated according to Equation (9), where $x=\varepsilon_{1}^{\pm }/\varepsilon_{k}^{\pm }$, $x=\varepsilon_{N}^{\pm }/\varepsilon_{k}^{\pm }$.

(12) $$d_{0}^{\pm }=A_{3}^{\pm }{\left(-\frac{n/N^{\pm }}{n/N^{\pm }-A_{4}^{\pm }}\right)}^{\frac{1}{A_{5}^{\pm }}}$$

where *n* and $N^{\pm }$ are the fatigue loading times and the fatigue life of concrete, respectively. The fatigue life of concrete is calculated according to Equation (13). The values of parameters $A_{3}^{\pm }$, $A_{4}^{\pm }$ and $A_{5}^{\pm }$ can be obtained from the literature [32].

(13) $$\log N^{\pm }=14.7-13.5\frac{\sigma_{\max}^{\pm }-\sigma_{\min}^{\pm }}{f_{k}^{\pm }-\sigma_{\min}^{\pm }}$$

where $\sigma_{\min}^{\pm }$ and $\sigma_{\max}^{\pm }$ are the maximum and minimum fatigue stress, respectively, and $f_{k}^{\pm }$ is the concrete’s tensile (compressive) strength.

The irrecoverable fatigue damage variable $d_{i,n}^{+}$ of concrete in the tension zone is given as [31]:

(14) $$d_{i,n}^{+}=\frac{d_{n}^{+}-d_{e,n}^{+}}{1-d_{e,n}^{+}}$$

(15) $$d_{e,n}^{+}=\left\{\begin{array}{ll} 0 & \varepsilon_{n}^{+}\leq 115.5\times 10^{-6}\\ 1-{\left(\frac{\varepsilon_{n}^{+}}{115\times 10^{-6}}\right)}^{-1.05} & \varepsilon_{n}^{+}\leq 115.5\times 10^{-6} \end{array}\right.$$

The irrecoverable fatigue damage variable $d_{i,n}^{-}$ of concrete in the compression zone is given as [31]:

(16) $$d_{i,n}^{-}=\left\{\begin{array}{ll} d_{n}^{-} & d_{n}^{-}\leq 0.2\\ \frac{1}{-5d_{n}^{-}+6} & d_{n}^{-}>0.2 \end{array}\right.$$

#### 3.1.2. Vibration Model of the Heavy-Haul Train

**Figure 14:**
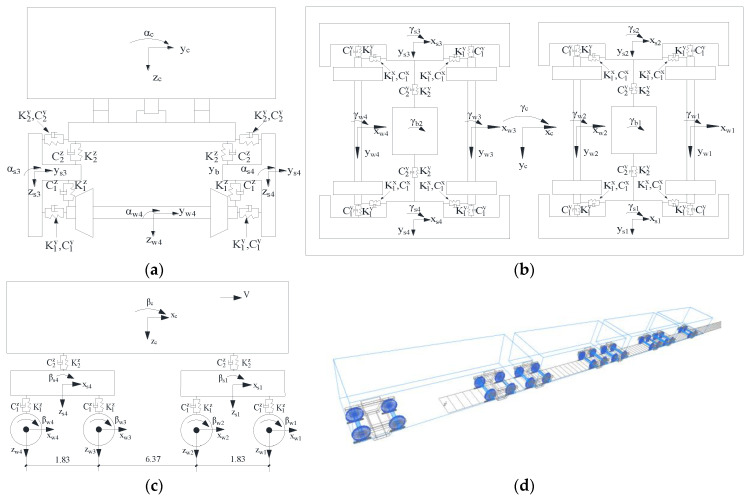
The vibration model of heavy-haul vehicle: (**a**) Front view of a vehicle; (**b**) Top view of a vehicle; (**c**) Side view of a vehicle (unit: m); (**d**) The finite element model of the heavy-haul train.

Figure 14 shows the model dimensions and component connections of heavy-haul trains. A train comprises one car body, two bolsters, four side frames, eight axle boxes, and four wheelsets. Among them, the car body, side frame, and wheelset are considered to have five degrees of freedom, namely, lateral movement, ups and down sways, shaking the head, side-rolling, and nodding. The wheelset and the bogie are connected by the primary suspension device. The secondary suspension device connects the car body and the bogie. The primary and secondary suspension devices are simplified to linear springs and dampers. In this paper, the finite element model of the heavy-haul train was established using the multi-body dynamics software SIMPACK [33], as shown in Figure 14d. The model size and mechanical parameters were selected according to the heavy-haul train C80 [34]. Through nominal stress calculation, the maximum residual acceleration of the train model system was taken as 1.796 × 10^−5^ m/s^2^, which is less than 0.01 m/s^2^, indicating that the balanced state of the train finite element model meets the requirements.

#### 3.1.3. Vibration Model of the Track-Bridge System 

**Figure 15:**
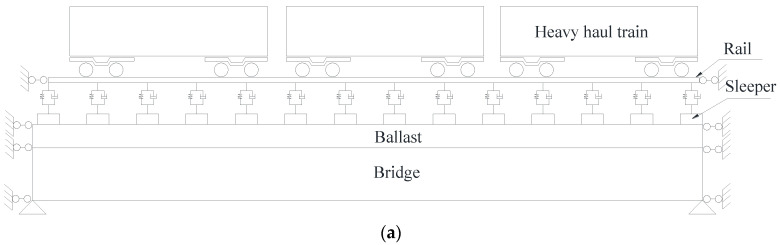

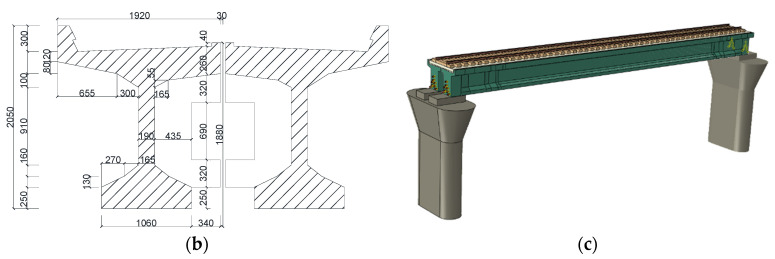
The finite element analysis model of the track-bridge: (**a**) The vehicle-rail-bridge system coupling model; (**b**) The cross-sectional form (unit: mm); (**c**) The finite element model.

The spatial vibration analysis model of the track-bridge, including rail, fastener, sleeper, beam, support, and pier, is shown in Figure 15a. The main girder is a 32 m standard height simply supported T-beam. A diaphragm is set between the two T-beams, and the cross-sectional form is shown in Figure 15b. The track-bridge is a multi-layer elastic discrete point support model [35], and its finite element model is shown in Figure 15c. The elastic modulus of the rail is 206,000 MPa; Poisson’s ratio is 0.3; and the density is 7.8 g/cm^3^. The elastic modulus of the sleeper is 32.5 MPa; Poisson’s ratio is 0.27; and the density is 2.5 g/cm^3^. The elastic modulus of the track-bed is 140 MPa; Poisson’s ratio is 0.27; and the density is 2.0 g/cm^3^. The parameters of concrete, tensile steel bars, and prestressing strand are consistent with those of the test beams.

### 3.2. Numerical Methods

#### 3.2.1. Fatigue Behavior Analysis Based on Vehicle-Rail-Bridge Coupling Vibration

**Figure 16:**
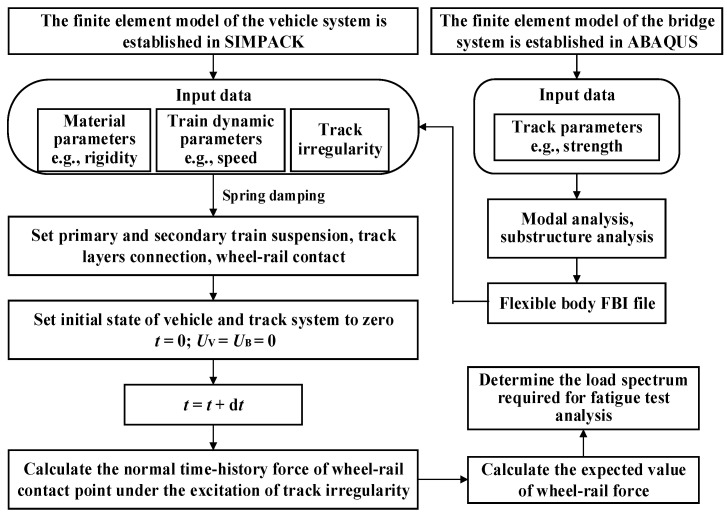
Fatigue load analysis based on vehicle-rail-bridge coupling vibration.

Figure 16 shows the fatigue behavior calculation flowchart for the prestressed concrete beams based on the coupling vibration analysis of vehicle-rail-bridge system. The analysis steps were as follows:

(1) The finite element model of the bridge was established in the finite element analysis software; the vehicle system dynamics model was established in SIMPACK [33].
(2) The modal analysis and substructure analysis of the bridge finite element model were performed, and the flexible body FBI files were generated and imported into SIMPACK [33].
(3) Spring damping was used to set up the track layers connection, and the primary and secondary suspension system of the train; the wheel-rail contact was used to realize the coupling effect of the track and bridge.
(4) The normal time-history force of wheel-rail contact point under the excitation of track irregularity was analyzed for trains with different axle weights. The expected values of wheel-rail force were calculated and applied to the finite element model in Figure 15. The stress of the tensile reinforcement was simulated to determine the fatigue test analysis load.

#### 3.2.2. Model Validation and Analysis 

**Figure 17:**
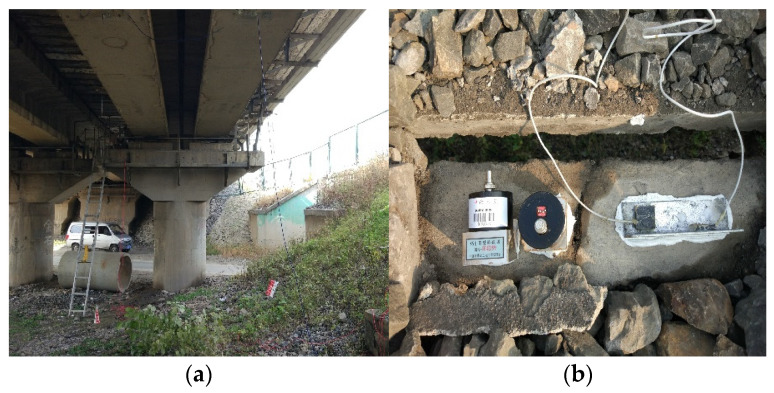
The layout of the field test sensors: (**a**) The layout of displacement sensors; (**b**) The layout of acceleration sensors.

A 32 m standard height prestressed concrete beam bridge of a heavy-haul railway was selected for a field test to verify the rationality of numerical calculation. When the C80 freight car (axle weight of 25 t) passed on the bridge at a speed of 60 km/h, the vertical amplitude and vertical acceleration response in the midspan of the beam were measured. The layout of field test sensors is shown in Figure 17. The displacement and acceleration sensors were arranged at the bottom of the beam and on the bridge deck, respectively. The simulation calculation was carried out for the same working conditions.

**Figure 18:**
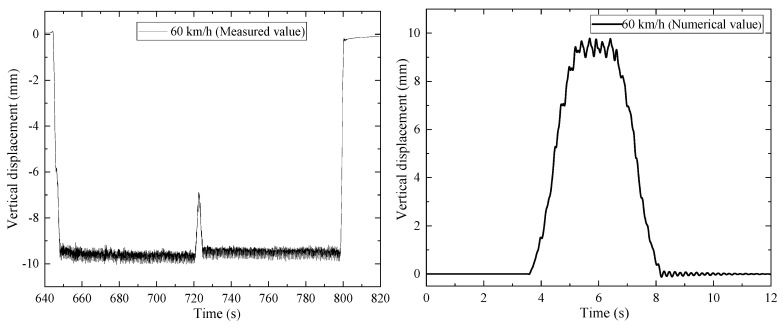
The midspan displacement time-history response of the beam.

Figure 18 shows the measured and simulated values of the midspan displacement time-history of a 32 m standard height prestressed concrete beam when the train passed at a speed of 60 km/h. There was a protruding section in the midspan vertical displacement between 720 s and 730 s of the measured value, which was the response as the locomotive in the train formation passed the bridge. The expected value of the time history displacement was 9.59 mm. The train adopted a 4-car formation in the simulation calculations, and a 5-level American track irregularity spectrum was applied. The expected value of the time history displacement of the bridge analysis model was 9.42 mm, and the error between the measured value and the simulated value was found to be 1.8%.

**Figure 19:**
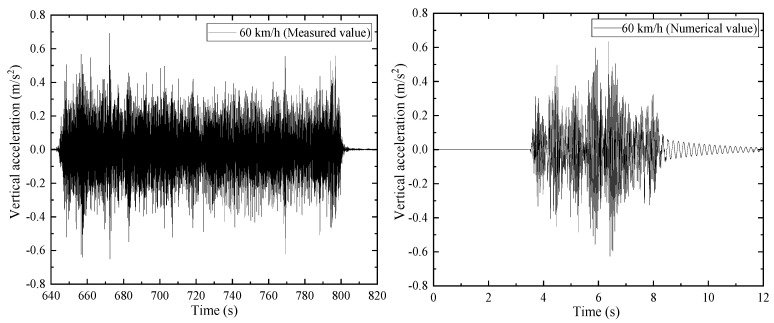
The midspan vertical acceleration time-history response of the beam.

Figure 19 shows the measured and simulated values of the midspan vertical acceleration time-history response of a 32 m ordinary height prestressed concrete beam when the train passed at the speed of 60 km/h. The peak response values of the measured and simulation results were found to be −0.65 m/s^2^ to 0.6921 m/s^2^ and −0.65 m/s^2^ to 0.6349 m/s^2^, respectively. The vertical acceleration simulation value was slightly different from the measured value. However, it was still also within the fluctuation range, indicating that the dynamic response of the bridge in the proposed vehicle-bridge coupling model can adequately reflect the actual dynamic characteristics of the structure.

**Figure 20:**
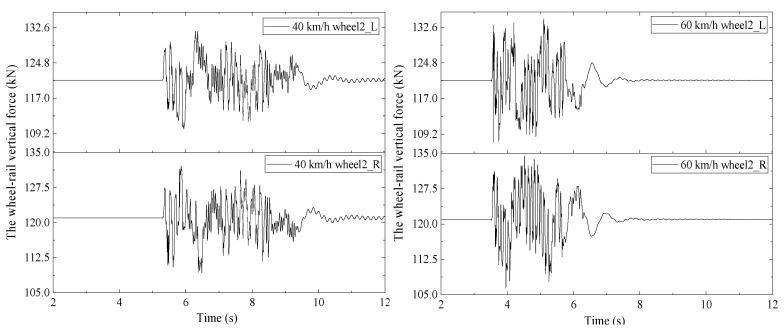
The wheel-rail force time-history curves.

The wheel-rail force time-history curves under different axle weights and operating speeds can be obtained through the vehicle-bridge coupling simulation calculation. Taking the second wheelset of the front bogie of the first train as an example, the vertical time-history force of wheel-rail is shown in Figure 20, when the train with 25 t axle weight passed through 32 m standard height prestressed concrete beam at speeds of 40 km/h and 60 km/h. Through the analysis step (4) in Section 3.1, the calculated stress amplitudes values of tensile reinforcement in the prestressed concrete beam were 45.93 MPa, 51.44 MPa, 55.11 MPa, and 60.62 MPa, when the train axle weights were 25 t, 28 t, 30 t, and 33 t, respectively. This can be used to determine the load of fatigue test analysis.

#### 3.2.3. Fatigue Behavior Assessment Model

**Figure 21:**
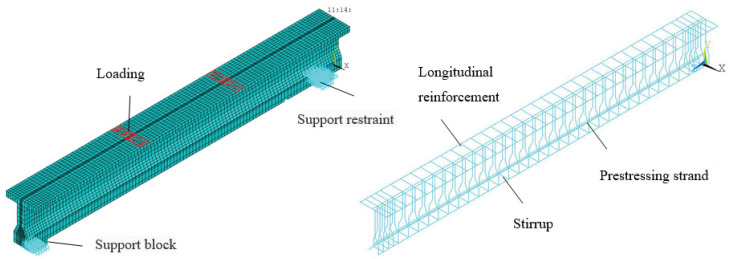
The finite element analysis model of prestressed concrete beam.

The finite element model of prestressed concrete beams is shown in Figure 21. The finite element model adopted the hexahedral element of type C3D8R, and line element of type T3D2, and the mesh size was 40 mm, with a total of 3766 element grids. The defined material parameters were consistent with the test beams. The cooling method was applied to prestress the beam (when the temperature drops, the steel strand will shrink and produce shrinkage stress).

The residual stiffness and yield point degradation of the beams loaded by 33 t and larger axle weight of trains were investigated, and analyzed the effects of train fatigue cycles and prestress loss on the residual stiffness and yield point degradation models of the beams.

The stress state of prestressed concrete beams before fatigue loading was calculated:

During the steel strand tensioning, the first batch of prestress loss (*σ*_lI_) occurs due to the friction between the prestressing strand and the pipeline, the anchor deformation, and the elastic shrinkage of concrete when the prestressed steel strand is tensioned in batches. After the prestressing strand is released, the concrete and non-prestressed steel bars experience pre-compression stress. The second batch of prestress loss of the prestressing strand (*σ*_l__II_) occurs due to the relaxation of the steel strands and the shrinkage and creep of the concrete, which include [36]:

(17) $$\sigma_{lI}=\sigma_{l1}+\sigma_{l2}+\sigma_{l4}$$

(18) $$\sigma_{lII}=\sigma_{l5}+\sigma_{l6}$$

In the above equation, *σ*_l1_ is the prestress loss caused by the friction between the prestressing strand and the pipeline. *σ*_l2_ is the prestress loss caused by the anchor deformation and the shrinkage of the prestressing strand. *σ*_l4_ is the prestress loss caused by the elastic shrinkage of concrete when the prestressed steel strand is tensioned in batches. *σ*_l5_ is the prestress loss caused by the relaxation of the prestressing strand, and *σ*_l6_ is the prestress loss caused by concrete shrinkage and creep. The prestress loss can be calculated according to the formulas given in the code [36]. The calculation parameters of effective prestress of prestressing strand before fatigue loading are shown in Table 4.

**Figure 22:**
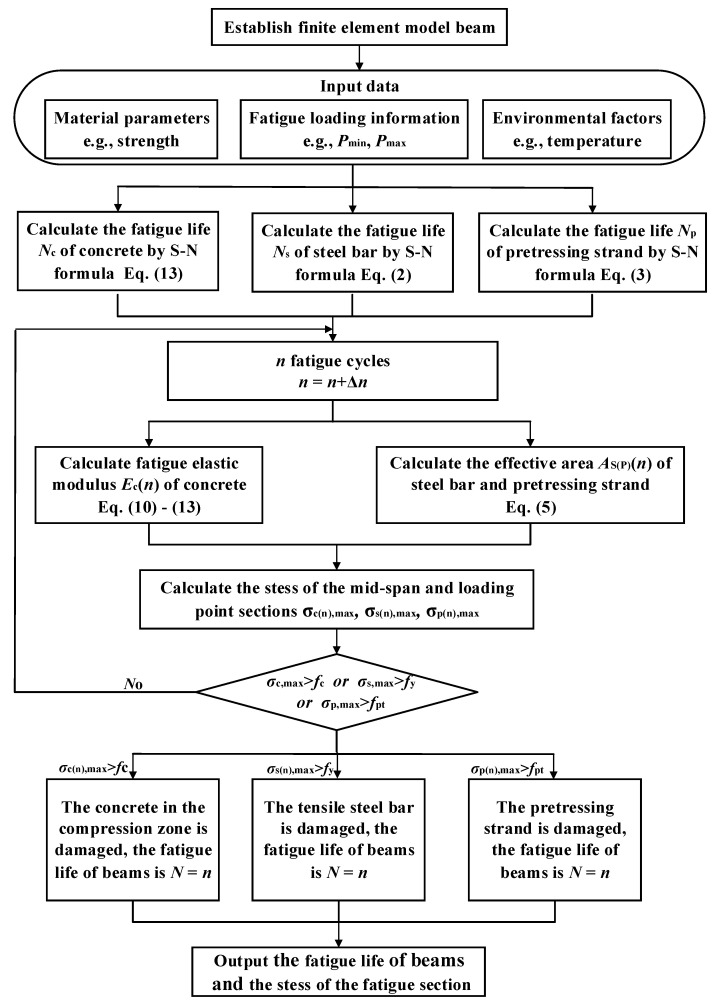
The fatigue behavior analysis process of prestressed concrete beams.

The finite element analysis method for evaluating the fatigue behavior of prestressed concrete beams is shown in Figure 22. The specific steps are as follows:

(1) The finite element model of prestressed concrete beams was established. The material parameters of concrete, prestressing strand, tensile steel bars, and stirrups were input. The material parameters were consistent with the test beams.
(2) The effective prestress of the prestressing strand before fatigue loading was calculated according to Equations (17) and (18). The initial prestress was applied to the prestressing strand by the cooling method.
(3) The stress amplitude of tensile steel bars, prestressing strand, and concrete in the compression zone under fatigue loading was determined. The fatigue life was calculated according to Equations (2), (3) and (13).
(4) After *n* fatigue cycles, the fatigue elastic modulus damage of concrete in the compression zone, the effective bearing area of the prestressing strand, and the tensile steel bars was calculated according to Equations (5) and (11). Then, the material properties were redefined.
(5) After *n* fatigue cycles, the maximum stress of concrete in the compression zone, prestressing strand, and tensile steel bars of beam midspan and loading point section was calculated.
(6) The damage of prestressed concrete beam was assessed by whether the concrete in the compression area, prestressing strand, and tensile steel bars reached the ultimate strength, i.e.,
  Yes: the fatigue life of prestressed concrete beams *N* = *n*.
  No: *n* = *n* + Δ*n*, return to step (4) and continue calculation.

#### 3.2.4. Validation through the Test Results

In the simulation calculation, the outermost tensile steel bars of prestressed concrete beams reached the ultimate tensile state first. When the tensile steel bars reached the ultimate tensile state, the prestressed concrete beams were regarded as damaged. The failure mode of the beam was consistent with that of the test beam. The tensile reinforcement fracture governs the collapse of the assessment beam. Hence, the relationship between the fatigue life of the prediction model and the stress amplitude of the tensile steel bars conforms to Equation (2). 

**Figure 23:**
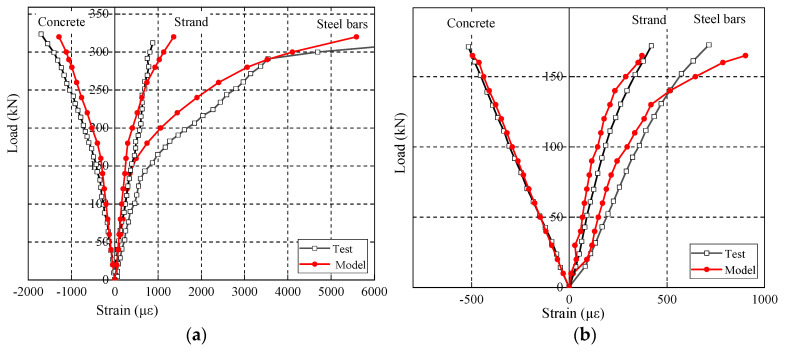
Finite element simulation results: (**a**) Load-strain curves of the S1 beam under static loading; (**b**) Load-strain curve of the C3 beam under fatigue loading.

The simulation results and test results of the beams S1 and C3 are compared in Figure 23 to verify the rationality of the assessment model. During the static loading process for beam S1, when the applied load was 168 kN, the maximum strain increase rate (the ratio of the strain increase value to the applied load range) of the tensile reinforcement increased significantly. The maximum tensile stress of the concrete in the tensile zone reached the tensile strength. When the applied load was 280 kN, the tensile reinforcement strain changed sharply and reached the yield strength of the reinforcement. At this time, the strain increase rate of concrete in the compression zone, and the prestressed steel strand increased significantly. The fatigue performance was verified for fatigue loading beam C3 by applying the maximum fatigue load and after 2.52 million cycles. The strain values of concrete in the compression zone, tensile steel bar, and prestressing strand were −515.88 µε, 714.76 µε, and 420.19 µε, respectively; the corresponding numerical calculation results were −494.91 µε, 902.00 µε, and 372.55 µε, with the error of 4.06%, 26.19%, and 11.33%. Therefore, the proposed fatigue behavior assessment model can effectively simulate the loading process of the beams. 

## 4. Model Application and Analysis 

A fatigue behavior analysis was conducted for the fatigue behavior assessment model by considering that the prestress loss was 0–30% and cracking coefficient was 0.85–1.23 during the fatigue process. The fatigue behaviors included fatigue life, stiffness, and yield capacity.

### 4.1. Fatigue Life of the Beam

**Figure 24:**
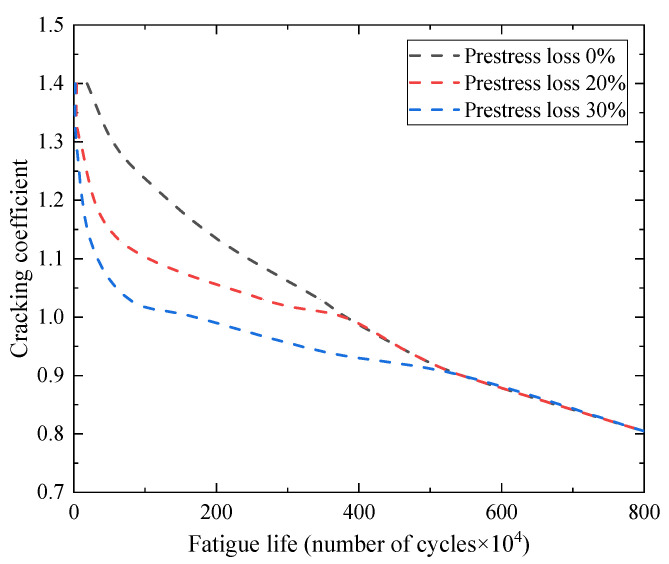
The effect of the fatigue load level and the prestress loss on the fatigue life of the beams.

The effect of the fatigue load level on the fatigue life of the beams is shown in Figure 24, where the cracking coefficient is the ratio of the maximum fatigue load to the cracking load. When the prestress loss was 0%, the cracking coefficient was 0.9, and the fatigue life of the beams was 5.44 million cycles. When the cracking coefficient increased from 0.9 to 1.03 and 1.13, the fatigue life values of the beams were 3.46 million cycles and 2.05 million cycles, respectively, which were reduced by 36.39% and 62.32%. The effect of prestress loss on the cracking load of the beams was not considered, and only the effect of prestress loss on the fatigue life of the beams under the same load level was analyzed. When prestress loss increased by 20%, and the cracking coefficient increased from 0.9 to 1.03 and 1.13, the fatigue life values of the beams were 2.66 million cycles and 0.65 million cycles, respectively, which were reduced by 51.10% and 88.05%. When prestress loss increased by 30%, and the cracking coefficient increased from 0.9 to 1.03 and 1.13, the fatigue life of the beams was 0.78 million cycles and 0.24 million cycles, respectively, which were reduced by 85.66% and 95.58%. When the maximum fatigue load exceeded the cracking load of the beam, the crack resistance of the fully prestressed concrete beam was poor, and the fatigue life of the beam decreased significantly with the increase in the fatigue load level and prestress loss. It can also be seen from the figure that when the maximum fatigue load was less than the cracking load, the prestress loss did not affect the fatigue life of the beam. This indicates that when the beam was not cracked, the prestressed concrete beam showed good fatigue performance, and the degree of prestressing had no effect on the fatigue life.

### 4.2. Stiffness Degradation

**Figure 25:**
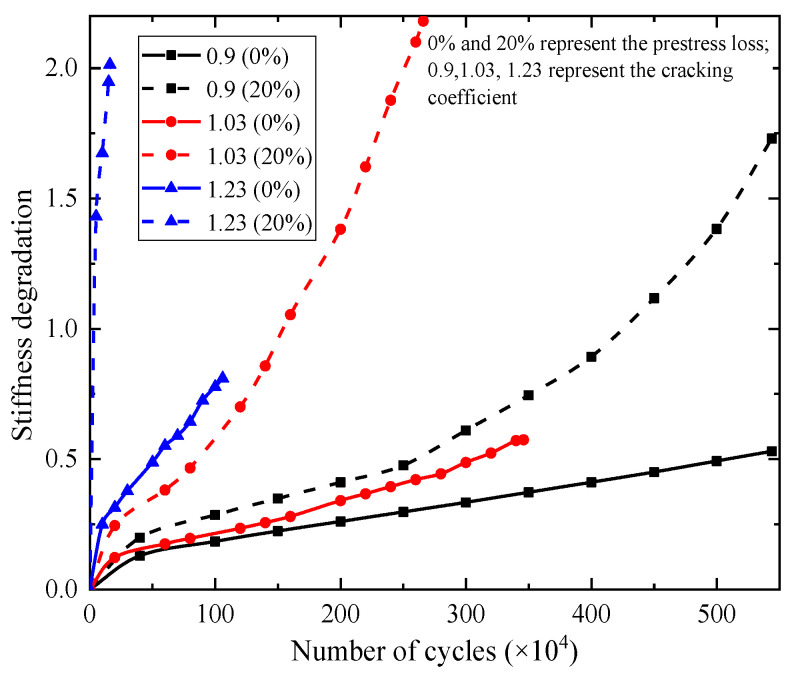
The effect of the fatigue load level and the prestress loss on the stiffness degradation of the beams.

The effects of the fatigue load levels and prestress loss on stiffness degradation of the beams are shown in Figure 25, where stiffness degradation coefficient is defined as α = (*f*_n_−*f*_0_)/*f*_0_, *f*_0_ is the midspan displacement before fatigue loading, and *f*_n_ is the midspan displacement after *n* fatigue cycles. When prestress loss was 0%, the cracking coefficient was 0.9, and the stiffness degradation degrees of the beams were 30%, 40%, and 50%, the corresponding fatigue cycles were 2.54 million cycles, 3.86 million cycles, and 5.10 million cycles, respectively. When the cracking coefficient increased from 0.9 to 1.03, the corresponding fatigue cycles were 1.73 million cycles, 2.44 million cycles, and 3.07 million cycles, respectively. When the cracking coefficient increased from 0.9 to 1.03 and prestress loss increased by 20%, the corresponding fatigue cycles were 0.36 million cycles, 0.64 million cycles, and 0.86 million cycles, respectively. The results show that the increase in the fatigue load level of the beams accelerated the stiffness degradation. The higher the degree of stiffness degradation, the more pronounced the influence of the fatigue load level on the stiffness. The concrete in the tension zone and tension steel bar gradually withdraw from work because of the accumulative fatigue damage. The fatigue capacity of the beams was borne mainly by the prestressing strand and the concrete in the compression zone. Prestress loss in the beams accelerated the degradation rate of the fatigue strength and reduced the fatigue life. The higher the fatigue damage degree, the more pronounced the effect of prestress loss on the fatigue life of the beams.

### 4.3. Post-Fatigue Strength

**Figure 26:**
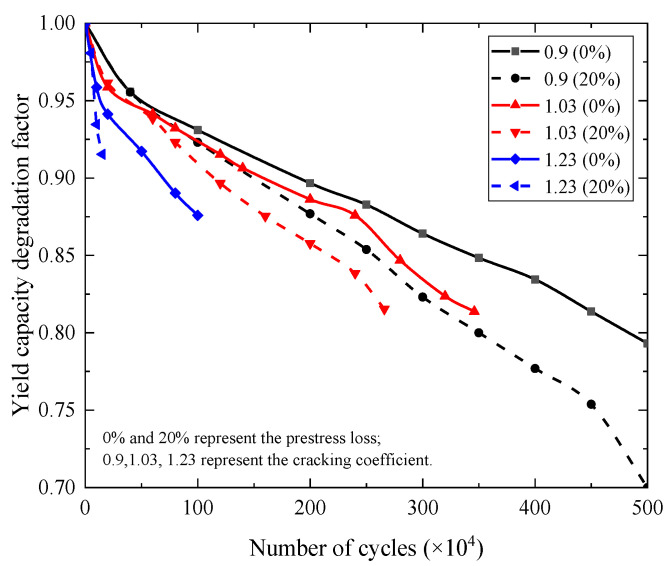
The effect of the fatigue load level and the prestress loss on the yield capacity degradation of the beams.

The impact of the fatigue load level on the yield capacity degradation of the beams is shown in Figure 26. The yield capacity degradation coefficient is defined as α_y_ = 1−(*P*_0_−*P*_n_)/*P*_0_, where *P*_0_ is the yield capacity of the beams before fatigue loading and *P*_n_ is the yield capacity after the beam is loaded for *n* times. It can be seen from the figure that the yield capacity degradation law of the beams was the same as that of the stiffness degradation. When prestress loss was 0%, the cracking coefficient was 0.9, and the yield capacity degradation values of the beams were 5%, 10%, and 15%, the corresponding fatigue cycles were 0.54 million, 1.90 million, and 3.45 million, respectively. When the cracking coefficient increased from 0.9 to 1.03, the corresponding fatigue cycles were 0.40 million, 1.59 million, and 2.76 million, respectively. When the cracking coefficient increased from 0.9 to 1.03 and prestress loss increased by 20%, the corresponding fatigue cycles were 0.40 million, 1.14 million, and 2.16 million, respectively. The results show that the increase in the fatigue load level and prestress loss in the beams accelerated the degradation rate of yield capacity. The higher the degree of yield capacity degradation, the more pronounced influence of the fatigue load level and prestress loss on the yield capacity. 

## 5. Conclusions

Due to the demand for increasing trainload and enhancing some existing heavy-haul railways, the low reserve value of bearing capacity is a problem for the 32 m-span simply supported beams. In this study, experiments were performed to investigate the fatigue behavior of prestressed concrete beams in a heavy-haul railway under 33 t and higher train loads, fatigue behaviors including fatigue deformation, fatigue life, crack propagation, and strains of classical materials. A fatigue behavior assessment model was established based on the experimental results to investigate the residual stiffness of the beams and yield point degradation under different prestress losses and load levels. The following conclusions can be drawn:

(1) When the trainload was lower than the cracking load and the beam was not cracked yet, the beams exhibited excellent fatigue performance, and the degree of prestressing will not affect the fatigue life. When the trainload exceeded the cracking load of the beam, the crack resistance of the prestressed concrete beam was poor, and the fatigue life of the beam decreased significantly with the increase in the trainload and prestress loss. The fatigue failure of prestressed concrete beams occurred in the bottom tensile steel bar. Therefore, it is reasonable to take the number of fatigue fracture cycles of tensile steel bars as the fatigue life of the beams.
(2) The crack development process of prestressed concrete beams displayed three stages: derivative stage, gradual development stage, and fatigue failure stage. Compared with partially prestressed concrete beams, fully prestressed concrete beams were more sensitive to cracks. Once cracked, the bottom tensile steel bar of the beams can easily undergo fatigue failure. Hence, the cracking of fully prestressed concrete beams should be strictly controlled.
(3) The static strength of prestressed concrete beams after fatigue loading was related to the fatigue damage degree. When the trainload was lower than the cracking load and the beam was not cracked yet, the degree of prestressing had little effect on the stiffness of the beam. When the beam was cracked with the accumulated fatigue damage or the trainload was greater than the cracking load of the beam, the concrete in the tension zone will gradually withdraw from work, and the force of the prestressed tendons will increase. Therefore, with the increase in the number of trainload fatigue cycles, the effect of prestress loss on the stiffness of the beam was more significant. With the increase in the trainload, the greater the fatigue damage degree of the prestressed concrete beam under the same fatigue cycles, and the greater the influence of prestress loss on the stiffness of the beam. Therefore, when the axle weight of the heavy-haul train is greater than the cracking load of beam, it is suggested to strengthen the beam by increasing prestress level.
(4) The increase in train load and prestress loss in the beams accelerates the degradation rate of yield-bearing capacity. The higher the degree of yield capacity degradation, the more pronounced the influence of fatigue load level and prestress loss on the yield capacity. When the trainload was lower than cracking load (cracking coefficient was 0.9) and the number of fatigue cycles was 3.45 million, the yield capacity of the beam decreased by 15%. Under the same degradation degree of yield capacity, when train load was greater than cracking load and the cracking coefficient increased from 0.9 to 1.03, the number of fatigue cycles of heavy-haul train decreased by 20%. The number of fatigue cycles decreased by 37.4% when the cracking coefficient increased from 0.9 to 1.03, and prestress loss increased by 20%. Therefore, when the prestressed concrete beam reaches a certain degree of fatigue damage, the axle weight of heavy-haul train should be strictly controlled to avoid yield failure of the beam.

## Figures and Tables

**Table 1:** Mixture ratio and type of concrete.

Mixture	Cement	Water	Sand	Aggregate
Mixture ratio (kg/m^3^) Types	495 P·II52.5R Portland cement	176 Tap water	648 Natural river sand	1152 Crushed limestone

**Table 2:** Material properties of reinforcement.

	Types	Nominal Diameter *d* (mm)	Yield Strength *f_y_* (MPa)	Ultimate Strength *f_u_* (MPa)
Longitudinal reinforcement	HRB335	10	405	475
Stirrup	Q235	8	310	455

**Table 3:** Fatigue test conditions of model beams.

Specimen Number	Applied Load at the Crack, Yield, and Ultimate, *P*_c_, *P*_u_ (kN)	Load Range (kN)		Load Level	Cracking Coefficient	Fatigue Life (×10^4^)
		*P*_min_	*P*_max_	*P*_max_/*P*_u_	*P*_max_/*P*_c_	
S1	157.0, 324.2	-	-	-	-	-
C1	-	11.42	136	0.42	0.85	-
C2	-	11.42	149	0.46	0.93	600
C3	-	11.42	165	0.51	1.03	301
C4	-	11.42	196	0.60	1.23	91

**Table 4:** The effective prestress of the prestressed concrete beam (unit: MPa).

The First Batch of Prestress Loss *σ*_lI_ = *σ*_l1_ + *σ*_l2_ + *σ*_l4_				The Second Batch of Prestress Loss *σ*_lII_ = *σ*_l5_ + *σ*_l6_			The Effective Prestress of Prestressing Strand
***σ*_l1_**	***σ*_l2_**	***σ*_l4_**	***σ*_lI_**	***σ*_l5_**	***σ*_l6_**	***σ*_lII_**	***σ*_pⅡ_ = *σ*_con_ − *σ*_lI_ − σ_lII_**
17.58	243.75	55.10	316.43	43.59	101.47	145.06	933.51

## Data Availability

Not applicable.
